# High Level of Aristolochic Acid Detected With a Unique Genomic Landscape Predicts Early UTUC Onset After Renal Transplantation in Taiwan

**DOI:** 10.3389/fonc.2021.828314

**Published:** 2022-01-06

**Authors:** Hong-Yue Lai, Li-Ching Wu, Po-Hsin Kong, Hsin-Hwa Tsai, Yen-Ta Chen, Yuan-Tso Cheng, Hao-Lun Luo, Chien-Feng Li

**Affiliations:** ^1^ Center for Precision Medicine, Chi Mei Medical Center, Tainan, Taiwan; ^2^ Department of Medical Research, Chi Mei Medical Center, Tainan, Taiwan; ^3^ Institute of Biomedical Sciences, National Sun Yat-sen University, Kaohsiung, Taiwan; ^4^ Department of Urology, Kaohsiung Chang Gung Memorial Hospital and Chang Gung University College of Medicine, Kaohsiung, Taiwan; ^5^ Center for Shockwave Medicine and Tissue Engineering, Kaohsiung Chang Gung Memorial Hospital and Chang Gung University College of Medicine, Kaohsiung, Taiwan; ^6^ Department of Clinical Pathology, Chi Mei Medical Center, Tainan, Taiwan; ^7^ National Institute of Cancer Research, National Health Research Institutes, Tainan, Taiwan; ^8^ Institute of Precision Medicine, National Sun Yat-Sen University, Kaohsiung, Taiwan; ^9^ Department of Pathology, School of Medicine, College of Medicine, Kaohsiung Medical University, Kaohsiung, Taiwan

**Keywords:** aristolochic acid, renal transplantation, UTUC, LC-MS/MS, mTOR, biomarker

## Abstract

**Background:**

The unusual high dialysis prevalence and upper urinary tract urothelial carcinoma (UTUC) incidence in Taiwan may attribute to aristolochic acid (AA), which is nephrotoxic and carcinogenic, exposure. AA can cause a unique mutagenic pattern showing A:T to T:A transversions (mutational Signature 22) analyzed by whole exome sequencing (WES). However, a fast and cost-effective tool is still lacking for clinical practice. To address this issue, we developed an efficient and quantitative platform for the quantitation of AA and tried to link AA detection with clinical outcomes and decipher the genomic landscape of UTUC in Taiwan.

**Patients and Methods:**

We recruited 61 patients with *de novo* onset of UTUC after kidney transplantation who underwent radical nephroureterectomy. A liquid chromatography-tandem mass spectrometry (LC-MS/MS) platform was developed for the quantitation of AA. Pearson’s chi-square test, Kaplan–Meier method, and Cox proportional hazard model were utilized to assess the correlations among AA detection, clinicopathological characteristics, and clinical outcomes. Seven tumors and seven paired normal tissues were sequenced using WES (approximately 800x sequencing depth) and analyzed by bioinformatic tool.

**Results:**

We found that high level of 7-(deoxyadenosin-N^6^-yl)aristolactam I (dA-AL-I) detected in paired normal tissues was significantly correlated with fast UTUC initiation times after renal transplantation (*p* = 0.035) and with no use of sirolimus (*p* = 0.046). Using WES analysis, we further observed that all tumor samples were featured by Signature 22 mutations, apolipoprotein B mRNA-editing enzyme, catalytic polypeptide (APOBEC)-associated gene mutations, *p53* mutations, no fibroblast growth factor receptor 3 (*FGFR3*) mutation, and high tumor mutation burden (TMB). Especially, mammalian target of rapamycin (mTOR) activation predominated in dA-AL-I-detected samples compared with those without dA-AL-I detection and might be associated with UTUC initiation through cell proliferation and suppression of UTUC progression *via* autophagy inhibition.

**Conclusion:**

Accordingly, dA-AL-I detection can provide more direct evidence to AA exposure and serve as a more specific predictive and prognostic biomarker for patients with *de novo* onset of UTUC after kidney transplantation.

## Introduction

Urothelial carcinoma (UC) occurs commonly (90–95%) in the urinary bladder (UB) but rarely (5–10%) in the upper urinary tract (UT) worldwide. Nevertheless, there is an unusual high incidence (>10%) of UTUC, including the renal pelvis and ureter, in Taiwan (1). End-stage renal disease (ESRD), also called kidney failure, is the last stage of chronic kidney disease (CKD), resulting in the need for long-term dialysis at a regular course. According to 2020 annual report of the United States Renal Data System (USRDS), Taiwan still ranks the first in terms of ESRD incidence and dialysis prevalence. Despite a long waiting time, kidney transplantation is regarded as the best treatment option for ESRD patients (2). However, the incidence of malignancy is higher in kidney recipients than in the general population (3). In contrast to the high incidence of skin cancer and lymphoma in Western countries (4), the incidence of UC in Taiwan has been reported to be high in kidney recipients (5). Collectively, the aberrant high UTUC incidence and dialysis prevalence in Taiwan suggest that the genomic landscape of UTUC in Taiwan is distinct from that in other areas and needs to be elucidated.

The extensive use of *Aristolochia* and *Asarum* and their related plants in traditional Chinese medicine has known to be associated with nephrotoxin and carcinogen exposure. Aristolochic acid (AA), a major bioactive component of these plants, has been reported to be a risk factor for CKD (6) and be correlated with the prevalence of UTUC in Taiwan (7). AA can be metabolized into aristolactam, which reacts with DNA to form covalent adducts with mutagenic properties (8). The predominant DNA adducts include 7-(deoxyadenosin-N^6^-yl)aristolactam I (dA-AL-I) and 7-(deoxyguanosin-N^2^-yl)aristolactam I (dG-AL-I) (9). Retrieving a record of 200,000 patients from National Health Insurance (NHI) in Taiwan, a study revealed that about one-third of the population in Taiwan had been exposed to AA between 1997 and 2003 (10). While the use of AA-containing herbal medication has been officially banned in Taiwan since 2003, aristolactam I adducts can be detected in renal tissues from patients with AA−induced nephropathy over 20 years after exposure to plants containing AA (11). Revealed by whole exome sequencing (WES), the mutational signature of AA is feature by A:T to T:A transversions (mutational Signature 22) located primarily on non-transcribed strand with a T/CAG sequence, particularly implicated in chromatin remodeling (12). Nevertheless, the WES analysis is not clinically feasible due to the high cost and time-consuming processes. Therefore, a fast and cost-effective approach such as liquid chromatography-tandem mass spectrometry (LC-MS/MS) for AA detection is warranted.

Immunosuppressive agents are crucial for suppression of allograft rejection but may predispose to the development of tumors (13). Sirolimus, also known as rapamycin, is a mammalian target of rapamycin (mTOR) inhibitor and has both immunosuppressive and anti-UC effects (14). Recently, rapamycin has also been reported to ameliorate AA−induced nephropathy through autophagy activation (15). However, autophagic activity has been indicated to be upregulated for UC cell proliferation (16), and autophagy inhibition can induce UC cell apoptosis (17). Accordingly, whether the use of sirolimus links to a better prognosis for kidney transplant UTUC patients remains an open question.

## Patients and Methods

### Patient Eligibility and Enrollment

This study was approved by the Institutional Review Board of Kaohsiung Chang Gung Medical Center (202000185B0). This study included 61 patients with *de novo* onset of UTUC after kidney transplantation who underwent radical nephroureterectomy from 2005 to 2019, and written informed consent was obtained from participants. Medication records, clinical and pathological features, and clinical outcomes were retrospectively obtained from the patients’ medical records. Our institutional follow-up protocol included postoperative fiber-cystoscopy every 3 months and renal ultrasonography to assess the contralateral urinary tract every 6 months during the first two years, every 6 months during the third year, and then annually thereafter. Computerized tomography (CT) scan of the abdomen was performed annually or depending on patients’ condition to assess lymph node status and local or regional recurrence of the tumor. Bone scans, chest CTs, and magnetic resonance images were performed when clinically indicated. Intravesical recurrence was defined as post nephroureterectomy urinary bladder tumor recurrence. Contralateral recurrence was defined as post nephroureterectomy contralateral upper urinary tract disease recurrence. Local recurrence was defined as locoregional recurrence at the ipsilateral surgical field, and distant metastasis was defined as disease recurrence outside urinary tract and out of the locoregional surgical field. Disease in the urinary bladder or contralateral upper urinary tract was not considered metastasis. Cancer-specific mortality was defined as local recurrence or distant metastasis at the time of death. The median and mean follow-up duration were 58.83 and 68.55 months, respectively.

### AA-Derived DNA Adduct Detection Platform Establishment

An LC-MS/MS platform (QTRAP 6500+, SCIEX) was developed for the quantitation of dA-AL-I and dG-AL-I, which are considered to be the most significant DNA adducts induced by aristolochic acids (9). The separation was achieved by reversed-phase liquid chromatography within 6 min, and their retention time were 2.11 min and 2.33 min for dG-AL-I and dA-AL-I, respectively. For the detection of dG-AL-I, the aglycone adduct (m/z 443) was selected to be the precursor ion because the N-glycosidic bond seemed to be unstable during ionization process. The fragments of dG-AL-I were observed at m/z 411 and m/z 293 due to the loss of methoxyl group and the cleavage of phenanthrene ring, respectively. For dA-AL-I, the protonated precursor ion at m/z 543 was detected, and its fragments at m/z 427 and m/z 292 corresponded to the loss of deoxyribose moiety and the cleavage of phenanthrene ring, respectively. Limit of detection (LOD) is usually considered acceptable when the signal-to-noise ratio (S/N) is greater than 3. In contrast, the S/N at limit of quantitation (LOQ) is usually greater than 10 and required to meet the criteria that inaccuracy and imprecision are lower than 20%. In our platform, the S/N of dA-AL-I and dG-AL-I were greater than 10 at the concentration of 10 pg/mL, and it passed the criteria of LOQ in the following validation process. Therefore, we set LOD and LOQ at the same concentration, equal to 1.2 adducts/10^8^ DNA bases when inputting 25 ug DNA.

### DNA Quality Check and Whole Exome Sequencing (WES)

The extracted genomic DNA (gDNA) was quantified using the Qubit fluorometer (Invitrogen) and the NanoDrop ND-1000 spectrophotometer (Thermo Fisher Scientific) following the manufacturer’s instructions. The quality check of DNA samples was also performed using the Agilent TapeStation system in combination with the genomic DNA ScreenTape assay, including the determination of DNA integrity number (DIN). As our specimens were from formalin-fixed paraffin-embedded (FFPE) tissues and about half tissues had stored for more than 10 years, we selected 7 specimens with high double-stranded DNA (dsDNA) concentration (> 600 ng) and high quality (DIN > 2.3) for further WES analysis.

A quantity of 200 ng dsDNA was used for the library construction. The DNA input was sheared by the Agilent SureSelectXT HS enzymatic fragmentation kit (5191-4080) and then constructed as a DNA library with unique molecular identifier (UMI). The UMI was used to reduce sequencing errors and PCR amplification bias. For the generation of standard exome capture libraries, the Agilent SureSelectXT low input reagent kit for Illumina multiplexed paired-end sequencing library protocol (G9703) was used with the SureSelect human all exon version 7 (48.2 Mb) probe set. The adaptor-ligated gDNA library (750 ng) was prepared for the hybridization with the probes, captured with the Dynabeads MyOne streptavidin T1 (Invitrogen), and then purified using the Agencourt AMPure XP beads (Beckman Coulter, Brea, CA, USA). The post-hybridization amplification was performed and analyzed with the Agilent D1000 ScreenTape assay on the TapeStation system. Finally, all libraries were sequenced on the Illumina NovaSeq 6000 sequencer with 2x150 paired-end sequencing protocol.

### Variant Calling and Mutational Signature Analysis

Sequencing data (FASTQ) were generated using bcl2fastq2 conversion software version 2.20. The raw FASTQ data were trimmed (read length < 30 bp) and filtered using Trimmomatic software version 0.36. Alignment of filtered reads against the analysis set of the GRCh38(hg38) reference genome was performed using BWA version 0.7.17. Furthermore, UMIs with specific molecular tag were matched using fgbio version 1.2.0. For duplicate removal, variant identification, normalization, multi-allelic splitting, and annotation, the genome analysis toolkit (GATK) version 4.1.1.0 and the variant effect predictor (VEP) version 100.2 were utilized. The variant filter was set at allelic depth (AD) ≥ 3 and on target regions. For somatic variant extraction, the paired normal tissue or tumor variants were subtracted from tumor or paired normal tissue variants, respectively. The extracted variants were classified as tumor-specific (somatically gained) or control-specific (somatically lost) variants and were subjected to the SigProfiler version 3.1 for mutational signature analysis. The global mutational signatures of each variant set were extracted from Catalogue Of Somatic Mutations In Cancer (COSMIC) single base substitution (SBS) signatures containing 96 different contexts (https://cancer.sanger.ac.uk/signatures/sbs/).

### Protein-Affecting Mutation (PAM) and Tumor Mutation Burden (TMB)

To identify the somatic mutations that alter original mechanisms, the protein-affecting mutations (PAMs) were defined as follows: i. the variants were located on RefSeq select or Ensembl canonical coding transcripts; ii. the variants were pathogenic/likely pathogenic in the ClinVar database (version 2020.03.29) or had maximal population allele frequency < 0.1% among the 1000 Genomes (1KG)/Genome Aggregation Database (gnomAD)/Trans-Omics for Precision Medicine (TOPMed) databases; iii. the variants were nonsynonymous (missense, nonsense, insertions, or deletions) or changed the codon sequence. For the tumor mutation burden (TMB) calculation, (cancer) driver genes were collected from the Oncology Knowledge Base (OncoKB) developed at Memorial Sloan Kettering Cancer Center (MSKCC) and COSMIC databases and the reference (18). The tumor-specific or control-specific PAMs were categorized into driver or non-driver panel. The TMB values of driver or non-driver genes of each patient were calculated by the number of tumor-specific or control-specific PAMs per megabase (Mb).

### Statistical Analysis

Pearson’s chi-square test was used to assess the correlations of AA-derived DNA adduct detection with clinicopathological characteristics. The Kaplan–Meier method with a log-rank test was applied to generate survival curves. To find independent prognostic factors, all significant parameters from the univariate analysis were put into the Cox proportional hazard model for multivariate analysis. All statistical analyses were conducted in SPSS software version 20.0 (IBM Corporation, Armonk, NY, USA), and two-tailed tests with a *p*-value < 0.05 were considered statistically significant.

## Results

### Clinicopathological Characteristics of Kidney Transplant UTUC Patients and Their Correlations With AA-Derived DNA Adduct Detection

**Figure 1:**
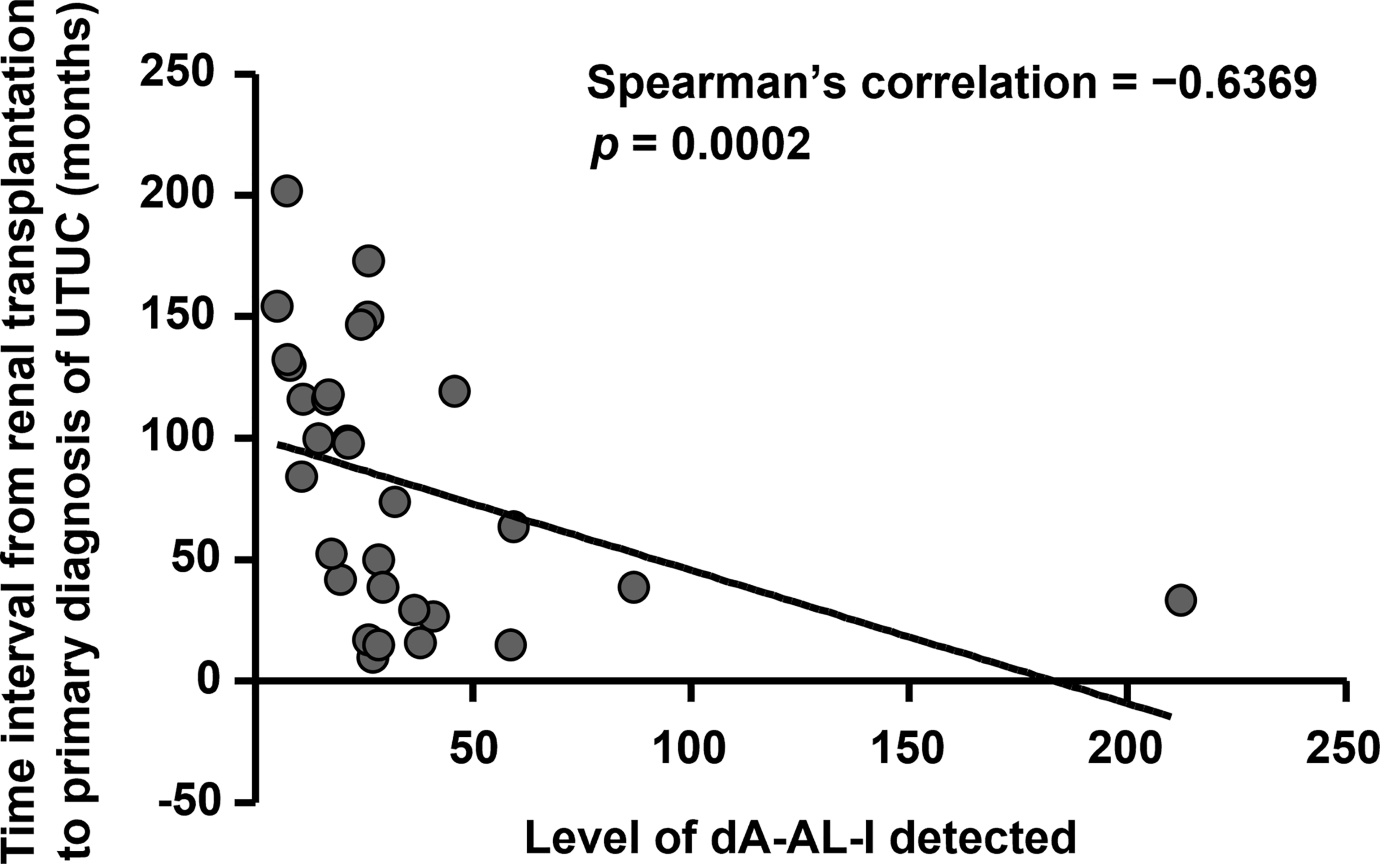
High level of dA-AL-I detected is significantly correlated with fast UTUC initiation times after renal transplantation. The level of dA-AL-I detected in paired normal tissues was performed by LC-MS/MS. The time interval from renal transplantation to primary diagnosis of UTUC was calculated. Spearman’s correlation was used to correlate the level of dA-AL-I detected with the UTUC initiation times after renal transplantation.

A total of 61 records of kidney transplant UTUC patients receiving radical nephroureterectomy were retrieved from our biobank, and most patients were female (*n* = 38, 62.3%) and under 60 years old (*n* = 44, 72.1%) (**Table 1**). The mean time interval between renal transplantation and primary diagnosis of UTUC was 79.7 months. To explore the correlations between AA-derived DNA adduct detection and clinical outcomes in UTUC, LC-MS/MS was conducted. As most tumor cells are featured by higher rates of proliferation and metabolism, we found that dA-AL-I could be detected in paired normal tissues (*n* = 31, 50.8%) instead of tumor samples. In addition, the dG-AL-I detection was observed only in paired normal tissue from 1 patient. Accordingly, we adopted dA-AL-I detection as an indicator of AA exposure in the following studies. High level of dA-AL-I detected in paired normal tissues was significantly correlated with a short period of time from renal transplantation to primary diagnosis of UTUC (*p* = 0.035) and with no administration of sirolimus (*p* = 0.046). As shown in **Figure 1**, the level of dA-AL-I detected in paired normal tissues was remarkably negatively correlated with the time interval from renal transplantation to primary diagnosis of UTUC (Spearman’s correlation = −0.6369, *p* = 0.0002).

**Table 1:** Correlations between AA detection and clinicopathological parameters in UTUC.

Parameter	Category	Upper Urinary Tract Urothelial Carcinoma				
		Case No.	AA detection (adducts/e8 DNA bases)			*p*-value
			ND	< 30	≥ 30	
Gender	Male	21	10	8	3	0.955
	Female	38	19	13	6	
Interval from renal transplantation to primary diagnosis of UTUC (months)	< 48	21	10	5	6	0.035*
	≥ 48, < 96	15	10	3	2	
	≥ 96	23	9	13	1	
Age (years)	< 60	44	24	12	8	0.068
	≥ 60	15	5	9	1	
Hepatitis B virus (HBV)	Negative	32	18	11	3	0.136
	Positive	13	4	5	4	
Hepatitis C virus (HCV)	Negative	45	23	15	7	0.422
	Positive	5	4	1	0	
Sirolimus (Rapamycin)	No use	26	8	13	5	0.046*
	Yes	35	22	9	4	
Cyclosporin A (CsA)	No use	25	10	11	4	0.47
	Yes	36	20	11	5	
Tacrolimus (FK506)	No use	13	9	3	1	0.262
	Yes	48	21	19	8	
Mycophenolate Mofetil (Cellcept)	No use	22	11	8	3	0.983
	Yes	39	19	14	6	
Renal pelvis tumor stage	Ta	6	4	2	0	0.058
	T1	9	4	1	4	
	T2-T4	19	14	4	1	
Ureter tumor stage	Ta	10	4	4	2	0.928
	T1	9	5	3	1	
	T2-T4	18	10	5	3	
Papillary	Absent	16	4	9	3	0.072
	Present	45	26	13	6	
High grade	Absent	2	0	2	0	0.16
	Present	59	30	20	9	
Lymphovascular invasion	Absent	50	23	18	9	0.279
	Present	11	7	4	0	
Squamous differentiation	Absent	46	21	18	7	0.61
	Present	15	9	4	2	
Carcinoma *in situ*	Absent	20	10	6	4	0.65
	Present	41	20	16	5	
Tumor necrosis	Absent	44	18	17	9	0.051
	Present	17	12	5	0	
Anemia (men < 13.5 g/dL, women < 12 g/dL)	Absent	28	12	13	3	0.26
	Present	30	16	8	6	
Neutrophil−lymphocyte ratio (NLR)	< 2	11	7	1	3	0.19
	≥ 2, < 5	19	11	7	1	
	≥ 5	14	8	2	4	
Body mass index (BMI)	< 24	38	21	11	6	0.079
	≥ 24	19	5	11	3	

ND, not detected; *, statistically significant.

The bold values are equal to statistically significant values.

### Survival and Prognostic Impact of Clinicopathological Factors in UTUC

At the univariate level (**Table 2**), males were considerably correlated with poor bladder recurrence-free survival (BRFS) (*p* = 0.008) and distant metastasis-free survival (DMFS) (*p* = 0.0138). Administration of sirolimus was considerably correlated with worse BRFS (*p* = 0.0188), whereas the use of cyclosporine was remarkably correlated with better DMFS (*p* = 0.0065). Advanced renal pelvic tumor stage was considerably correlated with better BRFS (*p* = 0.0174), while advanced ureteral tumor stage was significantly correlated with inferior local recurrence-free survival (LRFS) (*p* = 0.0414). Lymphovascular invasion (LVI) was remarkably correlated with poor LRFS and DMFS (both *p* ≤ 0.0008), whereas surrounding carcinoma *in situ* (CIS) was remarkably correlated with better LRFS and DMFS (both *p* ≤ 0.0014). Anemia was significantly correlated only with inferior LRFS (*p* = 0.0489). At the multivariate level (**Table 3**), males and early renal pelvic tumor stage were still unfavorable prognostic factors for BRFS (both *p* ≤ 0.011). Also, males and the presence of LVI remained independent prognostic factors for inferior DMFS (both *p* ≤ 0.046).

**Table 2:** Univariate log-rank analyses.

Parameter	Category	Case No.	OS		CRFS		BRFS		LRFS		DMFS	
			Event No.	*p*-value	Event No.	*p*-value	Event No.	*p*-value	Event No.	*p*-value	Event No.	*p*-value
Gender	Male	21	7	0.0944	5	0.755	14	0.008*	5	0.7457	9	0.0138*
	Female	38	7		10		14		8		6	
Interval from renal transplantation to primary diagnosis of UTUC (months)	< 48	21	6	0.4245	6	0.7511	14	0.0817	5	0.5676	7	0.8139
	≥ 48, < 96	15	2		5		7		2		3	
	≥ 96	23	6		4		7		6		5	
Age (years)	< 60	44	11	0.9763	11	0.754	20	0.682	10	0.915	10	0.3021
	≥ 60	15	3		4		8		3		5	
Hepatitis B virus (HBV)	Negative	32	7	0.6052	8	0.2739	12	0.1355	7	0.2489	8	0.4803
	Positive	13	2		5		8		1		2	
Hepatitis C virus (HCV)	Negative	45	7	0.0613	13	0.5236	20	0.4177	8	0.9546	9	0.4603
	Positive	5	3		2		3		1		2	
Sirolimus (Rapamycin)	No use	26	2	0.0976	7	0.5056	7	0.0188*	3	0.1449	6	0.754
	Yes	35	12		9		22		10		9	
Cyclosporin A (CsA)	No use	25	7	0.1077	6	0.6518	9	0.3815	7	0.2227	10	0.0065*
	Yes	36	7		10		20		6		5	
Tacrolimus (FK506)	No use	13	2	0.3953	5	0.3819	9	0.1252	3	0.9104	2	0.3637
	Yes	48	12		11		20		10		13	
Mycophenolate Mofetil (Cellcept)	No use	22	6	0.8788	4	0.1677	12	0.2592	5	0.9038	4	0.3515
	Yes	39	8		12		17		8		11	
Renal pelvis tumor stage	Ta	6	1	0.5632	3	0.8607	5	0.0174*	1	0.4047	1	0.6873
	T1	9	3		3		4		2		3	
	T2-T4	19	7		6		7		8		6	
Ureter tumor stage	Ta	10	2	0.1034	4	0.7836	5	0.4629	1	0.0414*	2	0.1207
	T1	9	0		2		8		0		1	
	T2-T4	18	7		5		11		7		9	
Papillary	Absent	16	5	0.0933	2	0.3319	5	0.1993	4	0.4546	5	0.2381
	Present	45	9		14		24		9		10	
High grade	Absent	2	1	0.1866	0	0.5052	0	0.2949	0	0.5264	0	0.5708
	Present	59	13		16		29		13		15	
Lymphovascular invasion	Absent	50	11	0.1885	14	0.9785	27	0.0739	7	0.0008*	7	< 0.0001*
	Present	11	3		2		2		6		8	
Squamous differentiation	Absent	46	10	0.834	14	0.2007	23	0.4272	11	0.4725	13	0.3095
	Present	15	4		2		6		2		2	
Carcinoma *in situ*	Absent	20	7	0.075	7	0.088	7	0.2201	10	0.0002*	10	0.0014*
	Present	41	7		9		22		3		5	
Tumor necrosis	Absent	44	8	0.1099	11	0.4739	24	0.1583	8	0.2131	10	0.4143
	Present	17	6		5		5		5		5	
Anemia (men < 13.5 g/dL, women < 12 g/dL)	Absent	28	5	0.2262	11	0.0555	12	0.3494	3	0.0489*	5	0.1275
	Present	30	8		4		16		9		10	
Neutrophil−lymphocyte ratio (NLR)	< 2	11	3	0.1641	2	0.2857	5	0.8145	4	0.45	4	0.1705
	≥ 2, < 5	19	2		5		11		3		2	
	≥ 5	14	5		5		7		4		5	
Body mass index (BMI)	< 24	38	7	0.5473	10	0.6329	16	0.2423	7	0.8807	8	0.3726
	≥ 24	19	5		4		11		3		6	
AA detection (adducts/e8 DNA bases)	ND	30	8	0.8428	10	0.4803	15	0.9644	8	0.6538	6	0.4608
	< 30	22	3		3		9		3		5	
	≥ 30	9	3		3		5		2		4	

OS, overall survival; CRFS, contralateral recurrence-free survival; BRFS, bladder recurrence-free survival; LRFS, local recurrence-free survival; DMFS, distant metastasis-free survival.

ND, not detected; *statistically significant.

The bold values are equal to statistically significant values.

**Table 3:** Multivariate survival analyses.

Parameter	Category	BRFS			LRFS			DMFS		
		HR	95% CI	*p*-Value	HR	95% CI	*p*-Value	HR	95% CI	*p*-Value
Gender	Male	1		0.011*	–	–	–	1		0.046*
	Female	0.24	0.08-0.72		–	–	–	0.329	0.111-0.979	
Sirolimus (Rapamycin)	No use	1		0.054	–	–	–	–	–	–
	Yes	3.75	0.977-14.403		–	–	–	–	–	–
Cyclosporin A (CsA)	No use	–	–	–	–	–	–	1		0.079
	Yes	–	–	–	–	–	–	0.36	0.116-1.123	
Renal pelvis tumor stage	Ta	1		0.005*	–	–	–	–	–	–
	T1	0.156	0.034-0.729		–	–	–	–	–	–
	T2-T4	0.073	0.015-0.354		–	–	–	–	–	–
Ureter tumor stage	Ta	–	–	–	1		0.931	–	–	–
	T1	–	–	–	0	0		–	–	–
	T2-T4	–	–	–	1.568	0.15-16.352		–	–	–
Lymphovascular invasion	Absent	–	–	–	1		0.065	1		0.009*
	Present	–	–	–	7.021	0.887-55.586		4.367	1.442-13.229	
Carcinoma *in situ*	Absent	–	–	–	1		0.156	1		0.058
	Present	–	–	–	0.292	0.053-1.598		0.325	0.102-1.039	
Anemia (men < 13.5 g/dL, women < 12 g/dL)	Absent	–	–	–	1		0.131	–	–	–
	Present	–	–	–	4.099	0.657-25.577		–	–	–

BRFS, bladder recurrence-free survival; LRFS, local recurrence-free survival; DMFS, distant metastasis-free survival.

*statistically significant.

The bold values are equal to statistically significant values.

### Molecular Characterization of Kidney Transplant UTUC Patients

To identify the molecular characterization of patients with *de novo* onset of UTUC after kidney transplantation, we sequenced 7 tumors and 7 paired normal tissues using WES analysis (approximately 800x sequencing depth). Among 7 paired normal tissues, 5 were detected with dA-AL-I (**Table 4**). The level of tumor mutation burden (TMB), defined as protein-affecting mutations (PAMs) per megabase (Mb), comprising driver and nondriver mutations was higher in tumors (all ≥ 65.5) compared with paired nontumor tissues (all ≤ 22.5), but these effects were not significantly different between dA-AL-I-detected and -nondetected specimens. Moreover, we found that all tumor samples bore aberrant apolipoprotein B mRNA-editing enzyme, catalytic polypeptide (APOBEC) cytidine deaminase activity with a higher frequency of mutations in chromatin regulation and DNA repair genes, such as lysine methyltransferase 2A (*KMT2A*), *KMT2C*, AT-rich interaction domain 1A (*ARID1A*), *TP53*, and ATRX chromatin remodeler (*ATRX*). Interestingly, 3 out of 5 tumor samples with dA-AL-I detected in paired normal tissues harbored mutations in *mTOR*, which serves as a cancer driver gene, at a higher frequency. Collectively, the AA mutational fingerprint can be observed in both tumor suppressor genes and oncogenes in AA-related UTUC.

**Table 4:** Correlations between AA detection and WES analysis.

AA Detection (Adducts/e8 DNA Bases)	Tumor Content (%)	WES (Normal)		WES (Tumor)		WES (Tumor)
		Nondriver TMB(Mutations/Mb)	Driver TMB(Mutations/Mb)	Nondriver TMB(Mutations/Mb)	Driver TMB(Mutations/Mb)	Driver genes
ND	80	6.6	12.4	42.3	44.9	ERCC2//JAK2//TBL1XR1//ATRX//COL5A1//SPTAN1//PTPDC1//PTPRD//SOX17//KEL//PIK3CG// EEF1A1//PIM1//HLA-B//NSD1//FBXW7//PDGFRA//PLXNB2//NF2//CASP8//PPM1D//KANSL1// BRCA1//NF1//NCOR1//TP53//CDH1//KMT2D//CHD4//CTNND1// MUC6//BTG2//EPHA2//
16.5	25	4.8	0	23.9	41.6	KMT2B//KMT2A//FLNA//STAG2//ZCCHC12//ATRX//AR//DMD//CDKN2A//CNBD1//FGFR1//KM T2C//HLA-B//MSH3//RFC1//NSD2//PLXNB2//CACNA1A//CREB3L3//AXIN2//KRT222//CDK12// NF1//TP53//ZFHX3//FLT3//MUC6//ELF3//MACF1//MTOR//
7.1	50	6.8	15.7	34.3	39.3	MSH6//KMT2A//SMARCA1//ATRX//USP9X//DMD//LEMD2//PIK3R1//PDGFRA//EP300//ASXL1// PCBP1//ARHGAP35//ZFHX3//NUP93//CHD8//KMT2D//INPPL1//MUC6//SPTA1//JAK1//THRAP3// ARID1A//
ND	50	6.1	9	58.2	73	VHL//KMT2A//STAG2//ATRX//ZMYM3//ABL1//SPTAN1//PTPRD//HGF//RAC1//MAP3K1//FAT1// POLQ//PBRM1//SCAF4//CUL3//ZFP36L2//CIC//CACNA1A//CD70//NF1//TP53//NUP93//CREBBP// TSC2//MGA//ATXN3//DACH1//BRCA2//ZMYM2//KMT2D//CHD4//MUC6//PTEN//RET//NUP133// BTG2//PTPRC//CSDE1//MACF1//ARID1A//EPHA2//
25.9	60	6	3.4	80.7	58.4	TAF1//ERBB3//TBL1XR1//ZMYM3//AMER1//RBM10//BCOR//DMD//RPS6KA3//PTPRD//KMT2C //HGF//GABRA6//FAT1//PIK3CA//RUNX1//FOXA2//ERBB4//PCBP1//XPO1//EPAS1//SOS1//ALK// APOB//CEBPA//CACNA1A//SMAD4//CDK12//CREBBP//RNF111//MGA//TRAF3//ZFP36L1//MAX //KMT2D//MUC6//RET//PTPRC//MACF1//MTOR//
37.9	40	7	5.6	51.9	68.5	MYH9//PRKAR1A//AMER1//HUWE1//DMD//COL5A1//TLR4//PTPRD//PIK3CG//GTF2I//EGFR//P MS2//CARD11//UNCX//ESR1//HLA-B//HLA-A//APC//NIPBL//ALB//RFC1//POLQ//SCAF4// ZNF133//KIF1A//ERBB4//APOB//EEF2//SMAD2//AXIN2//BRCA1//TP53//ZFHX3//NUP93//MAX// DACH1//LATS2//KMT2D//ATF7IP//CHD4//MUC6//SPTA1//NOTCH2//ARID1A//EPHA2//MTOR//
17.4	40	3.7	6.7	47.9	50.6	MYH9//DIAPH2//ATRX//AMER1//HUWE1//SMC1A//BCOR//COL5A1//SPTAN1//PTPRD//KMT2C //HGF//PIK3R1//FAT1//FBXW7//PBRM1//PLXNB2//SMARCB1//CUL3//APOB//SMAD2//SPOP//TP 53//CBFB//BRD7//TSC2//DICER1//CHD8//FLT3//KMT2D//ATM//RRAS2//MUC6//NUP133//ARID1A//

ND, not detected; WES, whole exome sequencing; TMB, tumor mutation burden.

### Mutational Signatures of Kidney Transplant UTUC Patients

**Figure 2:**
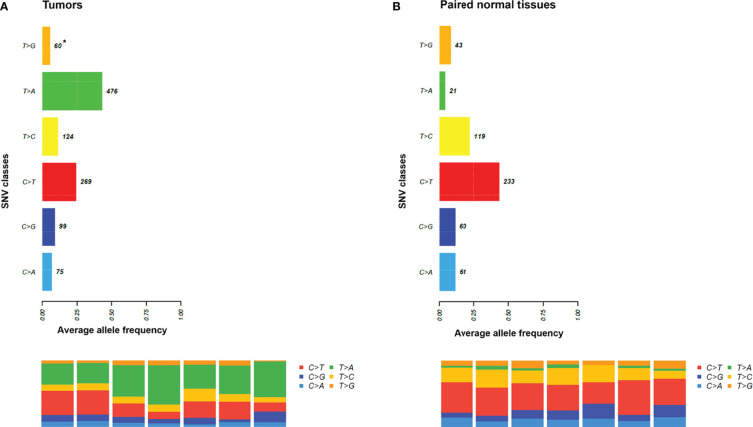
The pattern of single nucleotide variant (SNV) in tumors and paired normal tissues. **(A)** It was more likely to be T to A transversions in tumor samples. **(B)** It was more likely to be C to T transitions in paired normal tissues. *, average variant counts.

**Figure 3:**
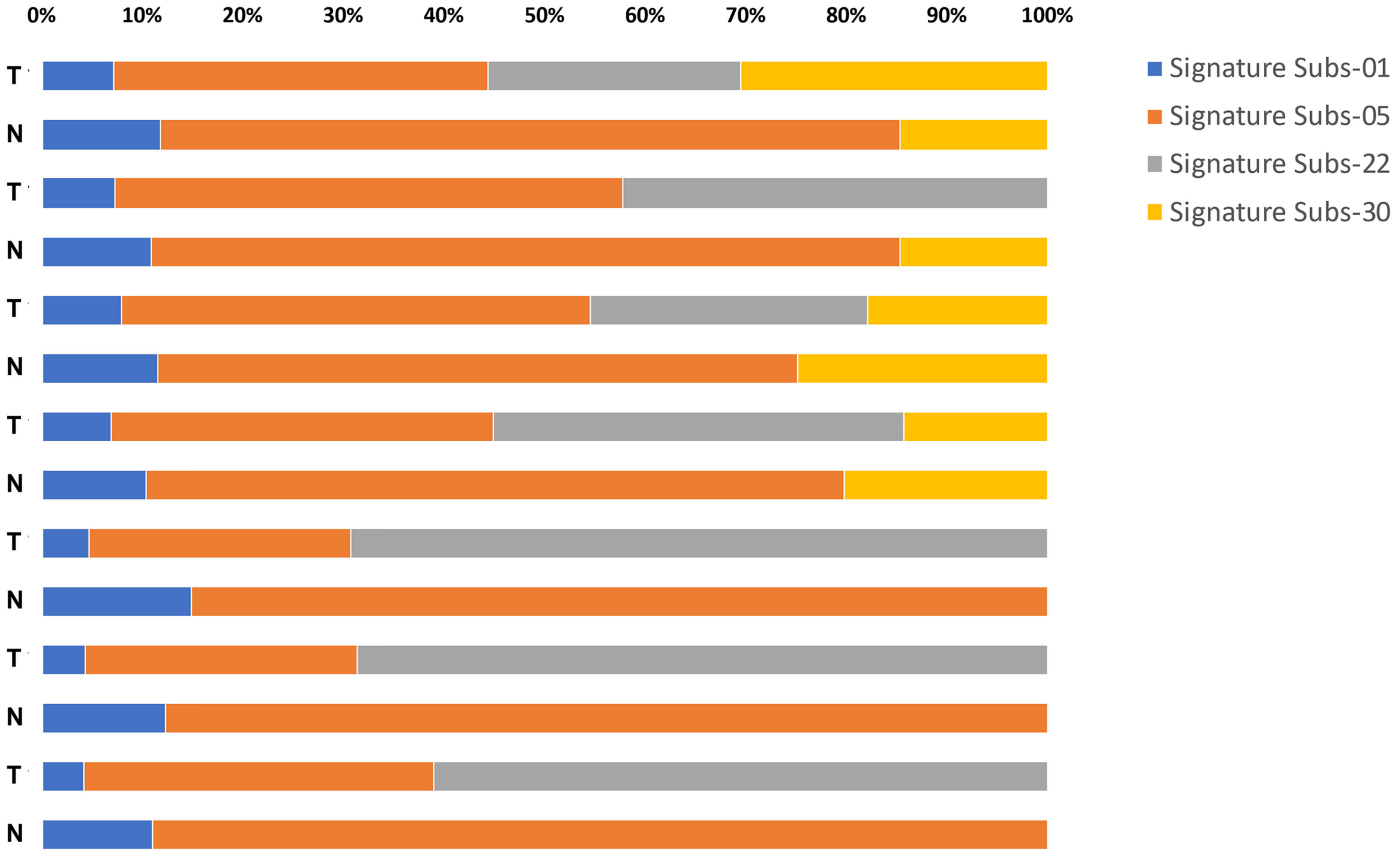
An SNV profile in tumors and paired normal tissues. Using nonnegative matrix factorization (NMF) analysis, the results showed that Signature 22 mutations were observed only in tumor samples regardless of whether dA-AL-I was detected in paired nontumor tissues or not.

**Figure 4:**
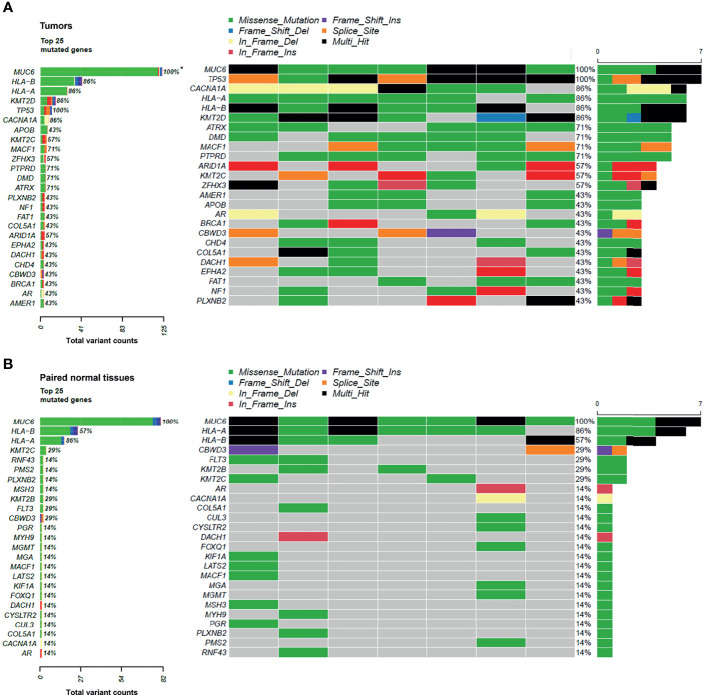
The top 25 mutated genes in tumors and paired normal tissues. **(A)** The top 25 mutated genes in tumor samples. **(B)** The top 25 mutated genes in paired normal tissues. *, number of mutated samples/seven samples (percentage).

Next, the mutational signatures determined by the Catalogue Of Somatic Mutations In Cancer (COSMIC) database were explored to show the dynamic crosstalk of risk factors and cellular processes during UTUC development. The pattern of single nucleotide variant (SNV) was more likely to be T to A transversions in tumor samples and C to T transitions in paired normal tissues (**Figures 2A, B**). Interestingly, the nonnegative matrix factorization (NMF) analysis further revealed that all tumor samples were featured by Signature 22 mutations regardless of whether dA-AL-I was detected in paired nontumor tissues or not, but there was no Signature 22 mutation identified in all paired normal tissues (**Figure 3**). **Figures 4A, B** display the top 25 mutated genes in tumors and paired normal tissues, respectively. Of these mutated genes, most were missense mutations.

## Discussion

With the advancement in sequencing technology, it has been suggested that aristolochic acid-induced COSMIC Signature 22 held great potential as a marker for aristolochic acid exposure (19) and favorable clinical outcomes (20) in UTUC. However, a more specific biomarker and cost-effective tool are still lacking. In this study, we developed an LC-MS/MS platform for the quantitation of AA. We found that high level of dA-AL-I detected in paired normal tissues was significantly correlated with early UTUC onset after renal transplantation and with no use of sirolimus (**Table 1**). Although we did not observe significant correlations between dA-AL-I detection and clinical outcomes, survival analysis revealed that no use of sirolimus was considerably correlated with better BRFS at the univariate level (**Table 2**), which suggests that dA-AL-I detection may be correlated with better BRFS. It has been indicated that UC incidence was higher in kidney recipients and that clinical outcomes for UC in kidney recipients were not worse than those without renal transplantation (3), and our observations may at least in part provide evidence that these phenomena may attribute to aristolochic acid exposure.

Additionally, combined with the molecular characterization, we noticed that dA-AL-I detection was more likely to be correlated with a higher frequency of *mTOR* mutations in tumor samples (**Table 4**). Aberrant activation of the mTOR pathway is crucial for tumor cell growth and proliferation. These may partially explain why the UTUC initiation times after renal transplantation was fast in patients detected with dA-AL-I. Nevertheless, we further found that, for kidney transplant UTUC patients with dA-AL-I detection, the use of sirolimus was remarkably correlated with inferior BRFS (*p* = 0.0068) (**Supplementary Figure 1**). As an mTOR inhibitor, sirolimus can activate autophagy, but it has been reported that autophagy is activated in UC and correlated with tumor progression (21). Since the level of autophagy is grade-dependent in UC, the mTOR activation (autophagy inhibition) may suppress tumor progression. Therefore, the associations among dA-AL-I detection, mTOR activation, autophagy level, and UTUC development deserve further investigation.

In our cohort, we found that *de novo* onset of UTUC after kidney transplantation was more common in females, while males served as an independent prognostic factor for inferior BRFS and DMFS at the multivariate level (**Table 3**). A recent study has indicated that there were more female patients in UTUC, but females were significantly correlated with better overall and cancer-specific survival in Taiwan (22), which shows similar results with our findings. In addition, we also noticed that advanced renal pelvic tumor stage remained a favorable prognostic factor for BRFS in the multivariate analysis. Following T2–T4 tumor stage-specific analysis, we further found that patients with coexistent renal pelvic/ureteral tumors or ureteral tumors alone seemed to have worse BRFS than those with renal pelvic tumors alone (*p* = 0.0199) (**Supplementary Figure 2**). Although the impact of initial tumor location on clinical outcomes in UTUC patients is still controversial (23, 24), our results provide evidence that renal pelvic tumors may be a favorable prognostic factor for kidney transplant UTUC patients.

Induced by multiple mutational processes, somatic mutations generate a characteristic mutational signature for each tumor (25). Over the past decade, an improved understanding of cancer genomes suggests common patterns representative of the sources of DNA damage that cause these observed mutations. Here, we found that tumor samples were featured by Signature 22 mutations even in dA-AL-I-nondetected samples (**Figure 3**). It has also been reported that mutational Signature 22 was observed in one sample of UTUC patients without previous indication of aristolochic acid exposure (19). These observations imply that Signature 22 mutations may be caused by unknown carcinogens, while the dA-AL-I detection platform we developed can provide more direct evidence to aristolochic acid exposure.

In addition to AA-induced Signature 22 mutations, one of the most distinguished mutational signatures to appear in UC is ascribable to the APOBEC family of enzymes (26). These enzymes act as single-stranded DNA cytosine deaminases and are involved in C>U deamination. The APOBEC mutational signatures are featured by Signature 2 and 13, which consist of C>T transitions and C>G transversions, respectively, occurring at cytosine nucleobases in 5’-TCW motifs (W = T or A) (25). We found that the counts of C>T transitions were not significantly different between tumors and paired normal tissues, but most of APOBEC-associated gene mutations were present at higher frequency only in tumor samples (**Figure 2** and **Table 4**). A study has also indicated that AA-related UTUC was characterized by APOBEC profile (27). Since there was no Signature 22 mutation identified in paired normal tissues in our study, whether Signature 22 mutations (T>A transversions) observed in tumor samples can enhance the frequencies of APOBEC-associated gene mutations needs further analysis. Moreover, it has been reported that mutational Signature 22 could be observed in both the APOBEC-low and -high groups (26), suggesting a more specific marker is needed. In this study, using LC-MS/MS, we demonstrated that dA-AL-I can be detected in paired normal tissues without mutational Signature 22 and APOBEC profile. Accordingly, dA-AL-I detected in normal tissues can serve as a useful biomarker applied to samples with low tumor content or early screening before tumor formation.

With an aggressive phenotype (28), UTUC has a distinct mutational profile as compared with UBUC. Immune checkpoint inhibitors (ICIs) are changing the way we treat UC, whereas the evidence concerning ICIs use in the management of UTUC remains scarce due to the rarity of the disease globally. The US Food and Drug Administration (FDA) has approved pembrolizumab (anti-PD-1) for the treatment of unresectable or metastatic TMB-high (≥10 mutations/Mb) tumors from any tumor histology, as determined by the FoundationOne CDx assay profiling 324 cancer-related genes. Accordingly, TMB has become a biomarker for ICIs, presuming that higher TMB will increase the number of neoantigens and specific T cell responses. Nevertheless, a recent study has suggested that high TMB may not predict the response to ICIs across all tumor types (29). In addition, an adaptive cutoff for TMB should take cancer types, sequencing depth, and sequencing technologies (panel-derived or WES-derived) into account to improve its predictive value (30). Using WES analysis (approximately 800x sequencing depth), we noticed that the estimated TMB comprising driver and nondriver mutations was higher in tumors (all ≥ 65.5) compared with paired nontumor tissues (all ≤ 22.5) regardless of whether dA-AL-I was detected or not (**Table 4**). Apart from TMB in tumors, the immunosuppressive microenvironment around the tumor may also affect the efficacy of ICIs (31). Autophagy has been suggested to promote growth of tumors with high TMB by limiting T cell immune responses (32), and autophagy inhibition can enhance antitumor immunity (33). Since the mTOR pathway was more likely to be activated (autophagy inhibition) in dA-AL-I-detected samples, whether dA-AL-I detection precisely predicts a better response to ICIs for UTUC with high TMB requires further investigation.

The current study has some restrictions. First, since germline mutations in DNA repair genes (34) and genomic scars of aberrant APOBEC enzymatic activity (35) may be correlated with bladder cancer risk and prognosis, the patient-matching peripheral blood mononuclear cells (PBMCs) should be utilized as a reference genome to define somatic mutations more accurately. Second, further experiments are required to validate the role of mTOR activation in AA-associated UTUC initiation and suppression of UTUC progression and the improved efficacy of ICIs. Finally, in this study, kidney transplant UTUC patients receiving radical nephroureterectomy were analyzed retrospectively at a single institution; consequently, the value of dA-AL-I detection should be prospectively verified by multi-center studies.

## Conclusion

In terms of UBUC, low-grade disease (~80%) is featured by hyperplasia, papillary phenotype, fibroblast growth factor receptor 3 (*FGFR3*) mutations, *HRAS* mutations, activation of the phosphatidylinositol-4,5-bisphosphate 3-kinase catalytic subunit alpha (PIK3CA)/AKT/mTOR pathway, and better outcomes, while high-grade disease (~20%) is characterized by dysplasia, carcinoma *in situ*, *p53* loss, *Rb* loss, and worse outcomes (36). In this study, we observed that UTUC samples with dA-AL-I detection were marked by Signature 22 mutations, APOBEC-associated gene mutations, *p53* mutations, mTOR activation, no *FGFR3* mutation, and high TMB. These observations imply that the mutational landscape of kidney transplant UTUC patients is more complex and distinct from that of UBUC patients, making the stratification of risk and clinical outcomes challenging. Accordingly, dA-AL-I detection can serve as a valuable predictive and prognostic biomarker for UTUC patients exposed to aristolochic acid.

## Data Availability Statement

The authors acknowledge that the data presented in this study must be deposited and made publicly available in an acceptable repository, prior to publication. Frontiers cannot accept a article that does not adhere to our open data policies.

## Ethics Statement

The studies involving human participants were reviewed and approved by Institutional Review Board of Kaohsiung Chang Gung Medical Center (202000185B0). The patients/participants provided their written informed consent to participate in this study.

## Author Contributions

Conceptualization, H-YL, L-CW, and C-FL. Methodology, P-HK, H-HT, Ye-TC, and Yu-TC. Investigation, H-YL, L-CW, and C-FL. Formal analysis, P-HK, H-HT, Ye-TC, and Yu-TC. Resources, H-LL and C-FL. Validation, H-YL, L-CW, P-HK, and H-HT. Visualization, H-YL, L-CW, P-HK, and H-HT. Writing - original draft, H-YL. Writing - review and editing, H-YL. Funding acquisition, H-LL and C-FL. Supervision: H-LL and C-FL. All authors contributed to the article and approved the submitted version.

## Conflict of Interest

The authors declare that the research was conducted in the absence of any commercial or financial relationships that could be construed as a potential conflict of interest.

## Publisher’s Note

All claims expressed in this article are solely those of the authors and do not necessarily represent those of their affiliated organizations, or those of the publisher, the editors and the reviewers. Any product that may be evaluated in this article, or claim that may be made by its manufacturer, is not guaranteed or endorsed by the publisher.

## Supplementary Material

Supplementary Figure 1 The use of sirolimus is remarkably correlated with inferior BRFS for kidney transplant UTUC patients with dA-AL-I detection. The Kaplan–Meier method with a log-rank test was applied to generate survival curves.

Supplementary Figure 2 Patients with coexistent renal pelvic/ureteral tumors or ureteral tumors alone have worse BRFS than those with renal pelvic tumors alone at T2–T4 tumor stage. The Kaplan–Meier method with a log-rank test was applied to generate survival curves.
