# Supplementary figures and images for: High Level of Aristolochic Acid Detected With a Unique Genomic Landscape Predicts Early UTUC Onset After Renal Transplantation in Taiwan
Source: Front Oncol. 2022 Jan 6;11:828314. doi: 10.3389/fonc.2021.828314 (PMC8770835; doi:10.3389/fonc.2021.828314)

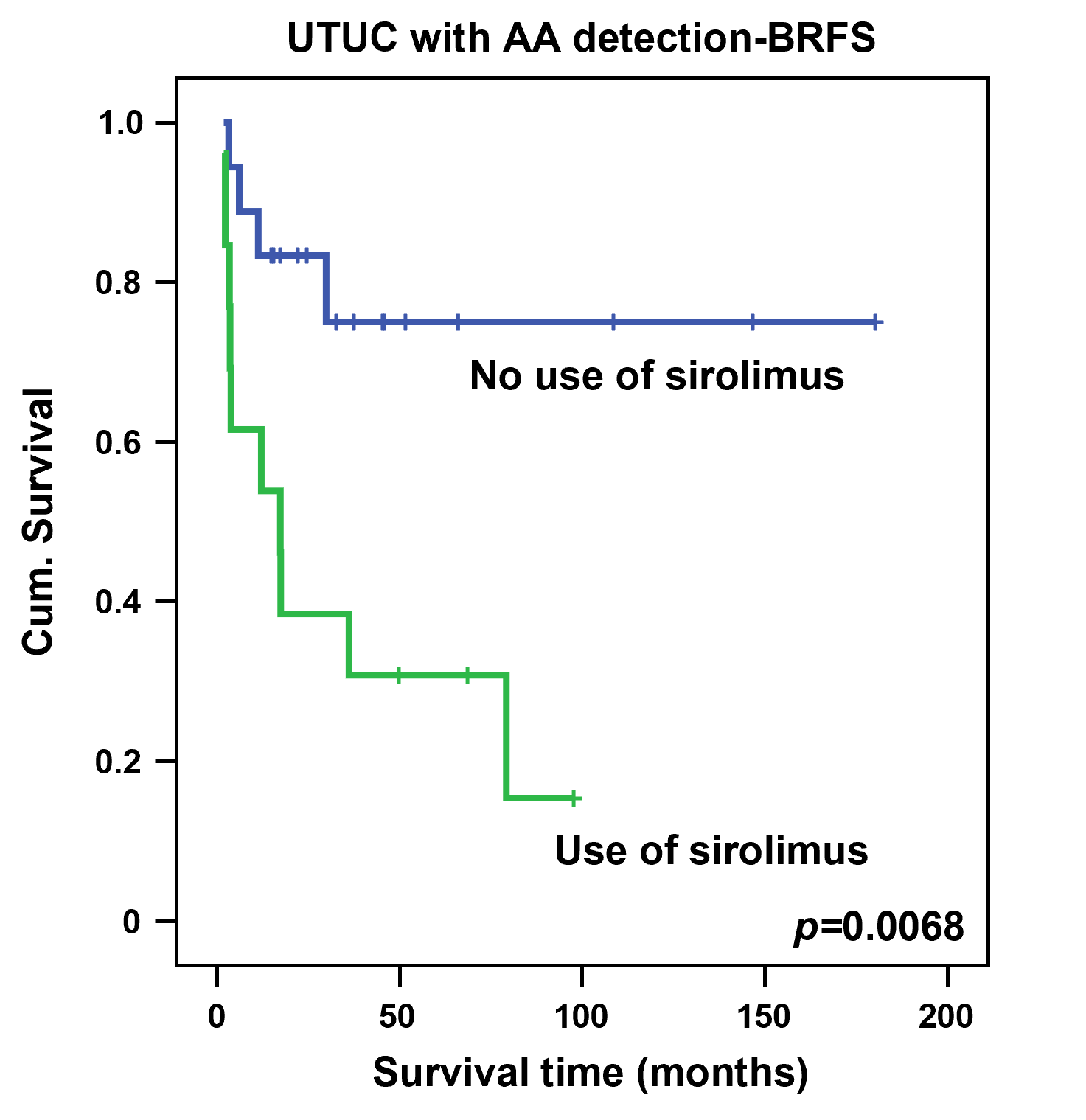

Supplement: Supplementary Figure 1 — The use of sirolimus is remarkably correlated with inferior BRFS for kidney transplant UTUC patients with dA-AL-I detection. The Kaplan–Meier method with a log-rank test was applied to generate survival curves. [file Image_1.tif]

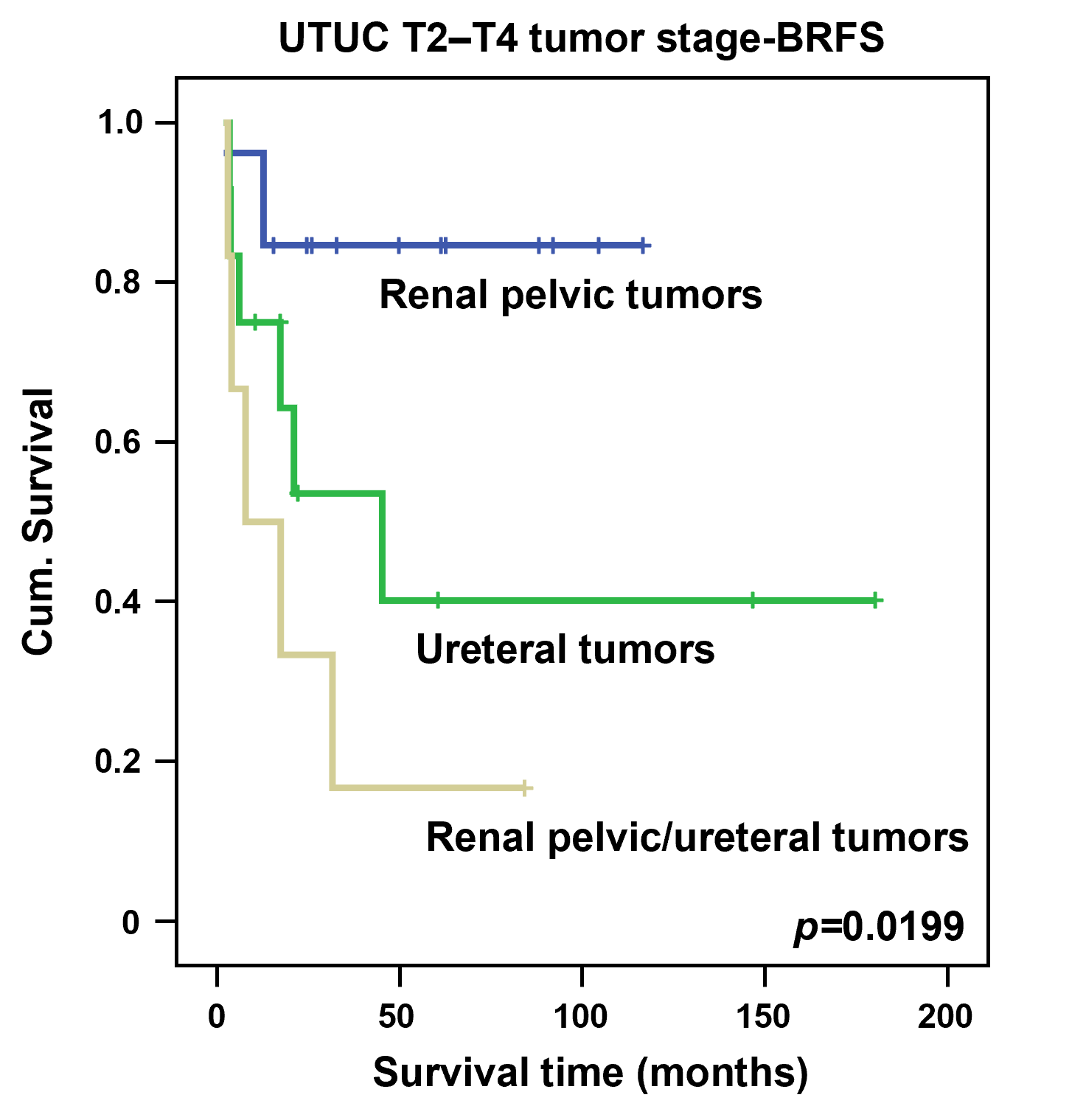

Supplement: Supplementary Figure 2 — Patients with coexistent renal pelvic/ureteral tumors or ureteral tumors alone have worse BRFS than those with renal pelvic tumors alone at T2–T4 tumor stage. The Kaplan–Meier method with a log-rank test was applied to generate survival curves. [file Image_2.tif]
